# Probiotics Alleviate the Progressive Deterioration of Motor Functions in a Mouse Model of Parkinson’s Disease

**DOI:** 10.3390/brainsci10040206

**Published:** 2020-04-01

**Authors:** Tsung-Hsun Hsieh, Chi-Wei Kuo, Kai-Hsuan Hsieh, Meng-Jyh Shieh, Chih-Wei Peng, Yen-Chien Chen, Ying-Ling Chang, Ying-Zu Huang, Chih-Chung Chen, Pi-Kai Chang, Kai-Yun Chen, Hsin-Yung Chen

**Affiliations:** 1 School of Physical Therapy and Graduate Institute of Rehabilitation Science, Chang Gung University, Taoyuan 33302, Taiwan; hsiehth@mail.cgu.edu.tw (T.-H.H.); kiwi725@gmail.com (C.-W.K.); katie911734@yahoo.com.tw (K.-H.H.); jackccchen@mail.cgu.edu.tw (C.-C.C.); 2 Neuroscience Research Center, Chang Gung Memorial Hospital, Taoyuan 33305, Taiwan; yzhuang@cgmh.org.tw; 3 Healthy Aging Research Center, Chang Gung University, Taoyuan 33302, Taiwan; 4 Department of Life Science, National Taiwan University, Taipei 10617, Taiwan; 5 Department of Biotechnology, Tajen Institute of Technology, Pingtung 90741, Taiwan; shieh@tajen.eduu.tw; 6 School of Biomedical Engineering, College of Biomedical Engineering, Taipei Medical University, Taipei 11031, Taiwan; cwpeng@tmu.edu.tw; 7 Department of Food and Nutrition, Taichung General Veteran Hospital, Taichung 40705, Taiwan; cychien@vghtc.gov.tw; 8 School and Graduate Institute of Traditional Chinese Medicine, College of Medicine, Chang Gung University, Taoyuan 33302, Taiwan; c1220@mail.cgu.edu.tw; 9 Division of Chinese Internal Medicine, Center for Traditional Chinese Medicine, Chang Gung Memorial Hospital, Taoyuan 33305, Taiwan; 10 Department of Neurology, Chang Gung Memorial Hospital and Chang Gung University College of Medicine, Taoyuan 33305, Taiwan; tyrant001dx@gmail.com; 11 Graduate Institute of Neural Regenerative Medicine, College of Medical Science and Technology, Taipei Medical University, Taipei 11031, Taiwan; kychen08@tmu.edu.tw; 12 Department of Occupational Therapy and Graduate Institute of Behavioral Sciences, College of Medicine, Chang Gung University, Taoyuan 33302, Taiwan; 13 Department of Neurology and Dementia Center, Chang Gung Memorial Hospital, Taoyuan 33378, Taiwan

**Keywords:** probiotics, Parkinson’s disease, neuroprotection, motor function, MitoPark, mice

## Abstract

Parkinson’s disease (PD) is one of the common long-term degenerative disorders that primarily affect motor systems. Gastrointestinal (GI) symptoms are common in individuals with PD and often present before motor symptoms. It has been found that gut dysbiosis to PD pathology is related to the severity of motor and non-motor symptoms in PD. Probiotics have been reported to have the ability to improve the symptoms related to constipation in PD patients. However, the evidence from preclinical or clinical research to verify the beneficial effects of probiotics for the motor functions in PD is still limited. An experimental PD animal model could be helpful in exploring the potential therapeutic strategy using probiotics. In the current study, we examined whether daily and long-term administration of probiotics has neuroprotective effects on nigrostriatal dopamine neurons and whether it can further alleviate the motor dysfunctions in PD mice. Transgenic MitoPark PD mice were chosen for this study and the effects of daily probiotic treatment on gait, beam balance, motor coordination, and the degeneration levels of dopaminergic neurons were identified. From the results, compared with the sham treatment group, we found that the daily administration of probiotics significantly reduced the motor impairments in gait pattern, balance function, and motor coordination. Immunohistochemically, a tyrosine hydroxylase (TH)-positive cell in the substantia nigra was significantly preserved in the probiotic-treated PD mice. These results showed that long-term administration of probiotics has neuroprotective effects on dopamine neurons and further attenuates the deterioration of motor dysfunctions in MitoPark PD mice. Our data further highlighted the promising possibility of the potential use of probiotics, which could be the relevant approach for further application on human PD subjects.

## 1. Introduction

Parkinson’s disease (PD) is one of the common age-related and progressive movement disorders [1,2]. Pathologically, PD is characterized by progressive degeneration of dopaminergic cells in the substantia nigra pars compacta (SNpc), leading to extrapyramidal motor dysfunctions, such as gait disturbance, resting tremor, rigidity, bradykinesia, and postural instability [2]. Typically, a diagnosis for the severity of PD is determined by the PD-related motor impairments. However, non-motor dysfunctions such as gastrointestinal (GI) dysfunctions (e.g., constipation, nausea, vomiting, gastroparesis, hypersalivation, dysphagia, delayed gastric emptying, defecatory anomalies, and pharyngoesophageal motor abnormalities found in manometric endoscopic diagnostics) are common symptoms affecting most patients with PD [3,4,5,6,7].

Earlier studies have reported the relationship between the brain and the GI system, the so-called gut–brain axis or brain–gut axis. It has been hypothesized that GI dysfunctions could reflect the disruptions of the microbiome–gut–brain axis, leading to serious GI inflammatory diseases (e.g., acute pancreatitis or inflammatory bowel disease), endothelial dysfunction, altered immune functioning and regulation of appetite control, neural inflammation, subsequent neurodegeneration, cognitive or psychoneurological disorders (e.g., depression, anxiety, autism, dementia), and disease progression of PD [8,9,10,11,12,13,14,15,16,17,18,19]. In addition to the use of peptides for the improvement of gastrointestinal or digestive dysfunctions [13,20,21,22], and in order to improve GI function and the balance of microbiota, probiotics could be one of the powerful tools to be used for altering the PD-associated microbiota composition and mitigating the related inflammatory process [12,23]. It could, therefore, inhibit the harmful gut bacteria and decrease the bacterial translocation, gut leakiness, and the associated neural inflammation in the enteric nervous system (ENS) [24,25,26,27]. Probiotics have been demonstrated as having potential for PD, including the improvement of stool consistency and bowel habits and the reduction of scores from the Movement Disorders Society-Unified Parkinson’s Disease Rating Scale (MDS-UPDRS) [28,29]. However, there is insufficient preclinical or clinical evidence to show the beneficial effects of probiotics in PD, and detailed underlying effects and mechanisms of probiotics in PD still need further clarification [30].

For translational research, the preclinical disease animal model could be helpful in providing a consistent condition to investigate the therapeutic effects of probiotics in PD. The current study was therefore designed to investigate the therapeutic potential of long-term administration of probiotics on a PD mouse model.

## 2. Materials and Methods

### 2.1. Animals

A genetic MitoPark PD mouse model was applied to the current study. The MitoPark PD mouse can exhibit the cardinal features of PD, such as the adult onset of degeneration in dopaminergic neurons and the progressive deterioration in motor functions [31,32]. Experiments were carried out on 16 male MitoPark PD mice. Eight-week-old PD mice were randomly assigned to the probiotic-treated group (*n* = 10) and sham treatment group (*n* = 10). Mice were group-housed with the same gender and given ad libitum access to water and food. The room temperature was maintained at 21–24°C, with a 12-hour light on/12-hour light off cycle. All animal procedures of this study were reviewed and approved by the Institutional Animal Care and Use Committee (IACUC) of Chang Gung University, Taoyuan, Taiwan (Approval number: CGU108-081, Period of Protocol: valid from Aug/01/2019-July/31/2022), and the Committee recognizes that the proposed animal experiment follows the guideline as shown in the Guide for Laboratory Animal Facilities and Care as promulgated by the Council of Agriculture, Executive Yuan, ROC (Taiwan). 

### 2.2. Probiotics

The experimental MitoPark PD mice were randomly assigned to two groups, namely the sham probiotic-treated group and the probiotic-treated group, to examine whether the daily administration of probiotics can alleviate motor dysfunctions. In the probiotic-treated group, each PD mouse involved in the treatment group received the probiotics (10^10^ CFU/mouse/day) dissolved in sterilized distilled water and mixed with Lieber–DeCarli liquid diet (Dyets, Inc. #D710027, Bethlehem, PA, USA) for 16 weeks (from the mouse age of 8 weeks to 24 weeks). The sham groups were given the regular Lieber–DeCarli all-liquid diet only without probiotics. The probiotics consisted of six bacterial strains (*Bifidobacterium bifidum*, *Bifidobacterium longum*, *Lactobacillus rhamnosis*, *Lactobacillus rhamnosus* GG, *Lactobacillus plantarum* LP28, and *Lactococcus lactis* subsp. *Lactis*) in a carrier matrix of maize starch, maltodextrins, and vegetable protein [27,33,34]. 

### 2.3. Behavioral Tests

**Figure 1:**
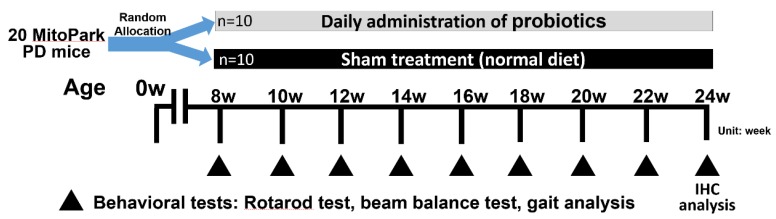
Experimental design for the identification of long-term effects of probiotic administration in MitoPark Parkinson’s disease (PD) mice (*n* = 20). Daily administration of probiotics was treated from the age of 8 weeks to 24 weeks in MitoPark PD mice. Behavioral tests, including rotarod, beam balance, and gait analysis, were performed every two weeks to test the time-course effects during treatment. Immunohistochemistry (IHC) analysis was applied at the age of 24 weeks to identify the neuroprotective effects on dopaminergic cells following probiotic intervention.

All behavioral tests were measured between 9:00 and 14:00. The tests were evaluated by the same rating device in a sound-attenuated room where the animals were acclimated for 30 min before the beginning of the tests. The apparatuses were cleaned with 95% ethanol between animals to avoid odor cues. Figure 1 shows the experimental design used in the present study. The rotarod, beam balance, and gait analysis tests were performed every two weeks after the intervention with or without probiotic treatment (Figure 1).

#### 2.3.1. Beam Balance Test

The beam balance test is used to detect the performance of motor skill and balance in the experimental rodent. The apparatus consisted of a plexiglass beam (120 cm long, 10 mm wide) and two vertical supports (30 cm high) above a square escape box (10 cm^3^ in area). A traditional lamp (with a 60-watt incandescent bulb) was applied to provide an aversive stimulus at the starting point [35]. All mice were trained to walk or run from a bright light point toward the dark home cage by traversing a beam at least four times before formal recording [36,37]. In each trial, the performance of the mouse was videotaped and then the average duration of the elapsed time to traverse the beam within five trials was calculated [37,38].

#### 2.3.2. Rotarod Testing

A rotarod test is a suitable tool for evaluating motor coordination and balance function [2,39,40]. The duration to balance on the rod was recorded at different rotational accelerations using a five-lane rotarod device (76-0771, Panlab Harvard Apparatus, Barcelona, Spain). For the rotational acceleration, the rotarod device was set to accelerate from 1 rpm to 30 rpm within 90 s. Five trials were measured in each mouse with a 15 min interval between trials [41].

#### 2.3.3. Gait Analysis

The procedures of gait analysis to measure the gait disturbance in PD mice were described previously [42,43]. The walking track was composed of an enclosed walkway made of transparent plexiglass (80 cm (length) × 6 cm (width) × 12 cm (height)) with a 45° tilting mirror positioned underneath the walkway. With a high-resolution and high-speed camera (PX-100, JVC, Yokohama, Japan) recording and image processing software (MathWorks, version 7.6., R2008a, Natick, MA, USA), several gait indices, including step length, stride length, step width, walking speed, and stance/swing phase time, were calculated [42,43,44].

### 2.4. Histology Investigation

After movement and behavioral tests, animals were sacrificed for tyrosine hydroxylase (TH) staining to detect the degeneration level of dopaminergic neurons in the SNpc. Briefly, mice were deeply anesthetized by an overdose of inhaled isoflurane with oxygen (0.1–0.3 L/min, 1.5%–3.5%). Then, mice were perfused intracardially with cold PBS solution and 4% paraformaldehyde fixative solution (PFA). Brains were then carefully removed, post-fixed, and cryoprotected in 30% sucrose solution at 4 °C for three days until the brain sank. The brain tissues were sectioned into slices of 30 um thickness on a cryostat (Leica CM3050 S, Buffalo Grove, IL, USA). The free-floating 30 um sections were quenched with 0.3% H_2_O_2_/PBS for 10 min, 10% milk (Anchor Shape-Up, Taipei, Taiwan) for 60 min to block non-specific antibodies. Next, the sections were incubated with 1:1000 rabbit primary anti-TH (AB152, Millipore, Burlington, MA, USA) at room temperature for 1 hour. Afterward, sections were washed in PBS and then incubated with the secondary 1:200 anti-rabbit antibody (MP-7401, Vector Labs, Burlingame, CA, USA) in PBS for 1 hour. After rinsing, immunostaining sections were visualized with 3,3′-diaminobenzidine (DAB) (SK-4105, Vector Labs) for 3–5 minutes. Then, the sections were mounted onto slides and air-dried. Finally, the sections were further dehydrated using xylol (Sinopharm, Shanghai, China) and cleared in xylene and cover-slipped in DPX [45]. The TH-positive neurons in the SNpc were counted manually in each section with a higher magnification (200×) microscope (Leica aperio scanscope C5, Buffalo Grove, IL, USA). TH-positive cells in the SNpc were counted using Aperio microscope Spectrum software (Aperio ScanScope CS, Vista, CA, USA).

### 2.5. Statistical Analysis

The effects of probiotics for all behavioral tests were assessed by two-way repeated measures ANOVA with groups (sham treatment group and probiotic treatment group) as a between-subjects factor and time (at different time points) as a within-subject factor. When the ANOVA showed a significant main effect of time, multiple comparisons between different time points were taken with the Bonferroni post hoc analysis. The unpaired *t*-test was used to verify the inter-group difference. Data were analyzed using SPSS version 25.0 (IBM Corporation, Armonk, NY, USA). The significance level was set at *p* < 0.05. All data were showed as the mean ± standard error of the mean (SEM).

## 3. Results

### 3.1. Long-Term Probiotic Treatment Mitigates Beam Balance and Motor Coordination in MitoPark PD Mouse Model

**Figure 2:**
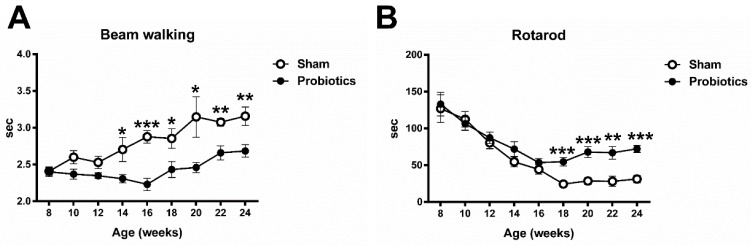
The effect of long-term probiotic administration on balance function (**A**) and motor coordination (**B**) evaluated by the beam walking test and rotarod test, respectively. The duration of the elapsed time for the probiotic-treated PD mice was significantly lower than that of the sham-treated PD mice after the age of 14 weeks. In the rotarod test, the latency for the probiotic-treated mice was significantly longer than that of the sham-treated mice after the age of 18 weeks. Values are presented as the mean ± SEM. * *p* < 0.05, ** *p* < 0.01, *** *p* < 0.001, significant differences between two groups.

The present study used the beam walking and rotarod test to investigate the performance in balance function and motor coordination of the MitoPark mice with or without probiotic administration in different age stages. In the beam walking test, repeated measures ANOVA indicated significant main effects of group (F = 18.72, *p* = 0.001) and time (F = 8.25, *p* < 0.001) and a significant interaction with group × time (F = 2.59, *p* = 0.013). Post hoc *t*-tests between the two groups showed that time duration reached a significant difference at the age of 14 weeks (t = 2.31; *p* = 0.039), 16 weeks (t = 5.38; *p* < 0.001), 18 weeks (t = 2.48; *p* =0.029), 20 weeks (t = 2.42; *p* =0.032), 22 weeks (t = 3.89; *p* =0.002), and 24 weeks (t = 3.11; *p* = 0.01) (Figure 2A).

For the performance of motor coordination using the rotarod test, repeated measures ANOVA indicated significant main effects of group (F = 11.32, *p* = 0.003) and time (F = 28.83, *p* < 0.001) and a significant interaction with group × time (F = 2.17, *p* = 0.033). The *t*-tests showed that time duration reached a significant difference at the age of 18 weeks (t = 4.33; *p* < 0.001), 20 weeks (t = 4.40; *p* < 0.001), 22 weeks (t = 3.52; *p* =0.002), and 24 weeks (t = 5.82; *p* < 0.001) between groups (Figure 2B).

### 3.2. Effect of Probiotics on Gait Pattern

**Figure 3:**
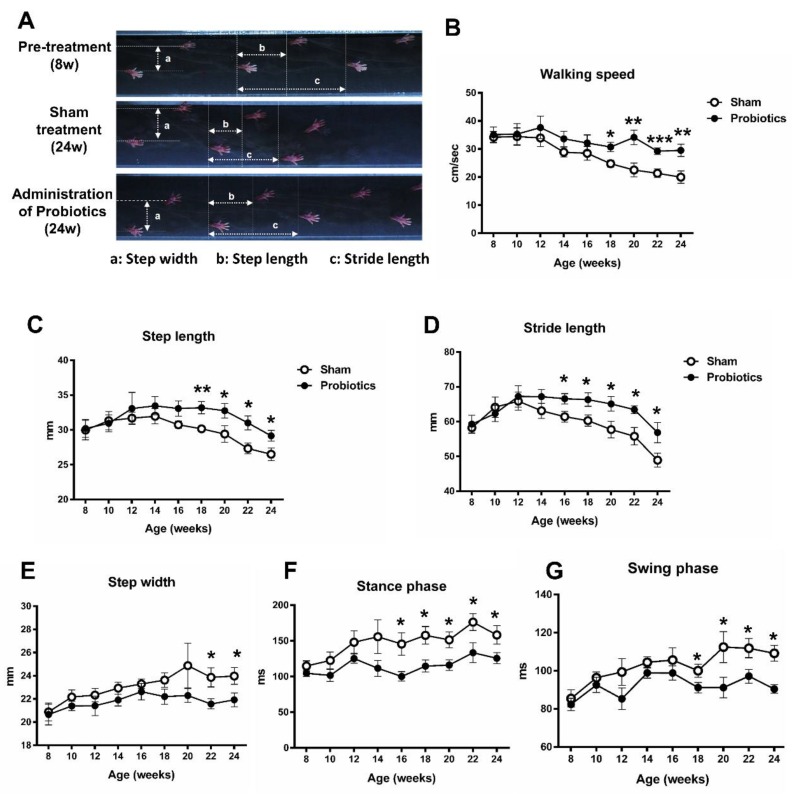
Characteristics of stepping footprints during a 2-sec walkway walking for an 8-week-old MitoPark PD mouse, a 24-week-old MitoPark PD mouse with non-probiotic treatment, and a 24-week-old MitoPark PD mouse with long-term probiotic treatment (**A**). Time-course changes in walking speed (**B**), step length (**C**), stride length (**D**), step width (**E**), stance phase time (**F**), and swing phase time (**G**) in the sham- and probiotic-treated PD mice over a 16-week observation period. The results showed that the gait function decreases significantly in the sham-probiotic-treated group but decreases less in the probiotic treatment group. * *p* < 0.05, ** *p* < 0.01, *** *p* < 0.001, significant difference between the two groups at each time point.

Figure 3A shows typical images of footprints recorded from a MitoPark PD mouse with sham treatment and a PD mouse with long-term administration of probiotics. Two-way repeated measures ANOVA revealed the significant main effects of group on walking speed (F = 6.12, *p* = 0.023), step length (F = 5.17, *p* = 0.035), stride length (F = 5.76, *p* = 0.027), step width (F = 6.75, *p* = 0.018), stance phase time (F = 11.94, *p* = 0.003), and swing phase time (F = 9.13, *p* = 0.007), suggesting less dysfunction in gait pattern in the probiotic-treated PD mice than in the sham-treated PD mice. The further *t*-tests between two groups showed that this difference was driven by probiotic treatment effects observed in walking speed, step length, stride length, step width, stance phase time, and swing phase time after 8–14 weeks of probiotic treatment (unpaired *t*-tests, *p* < 0.05) (Figure 3B–G).

### 3.3. Effect of Probiotic Treatment Tested by Immunohistochemistry (IHC) in PD Mice

**Figure 4:**
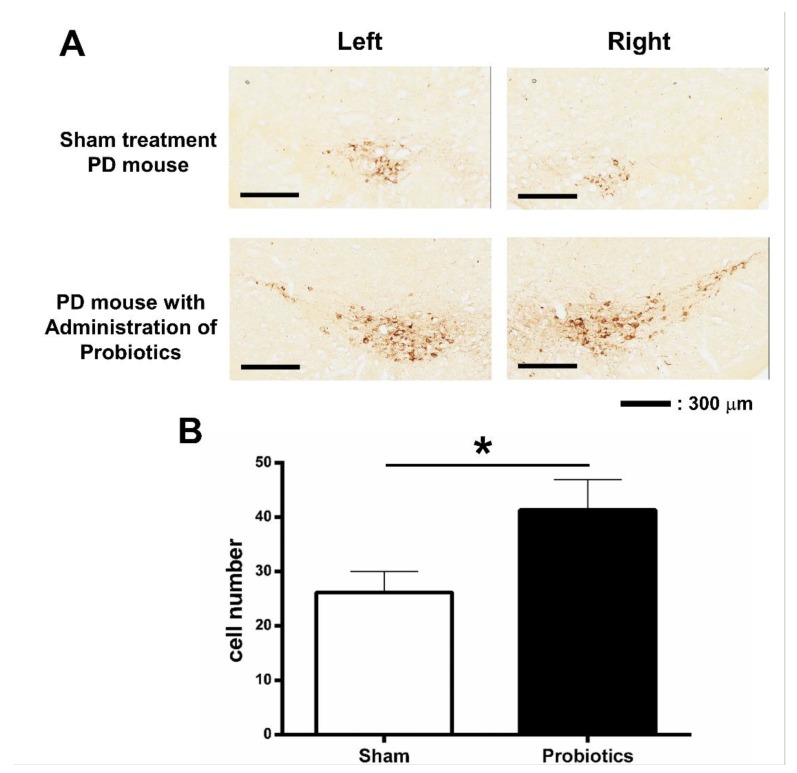
Representative tyrosine hydroxylase (TH) immunostaining in the substantia nigra pars compacta (SNpc) in PD mouse without probiotic treatment and in PD mouse with probiotic treatment (**A**). The average numbers of TH-positive neurons in the SNpc between sham treatment and probiotic-treated groups were compared (**B**). * *p* < 0.05 (unpaired *t*-test).

Regarding the effects of the 16-week probiotic treatment on dopaminergic cells in the SNpc, the results of TH immunohistochemistry are shown in Figure 4A. The TH-positive cells in the SNpc at the age of 24 weeks in PD mice are quantified in Figure 4B. PD mice receiving probiotic treatment showed preservation of TH-positive cells in the SNpc (t = 2.22, *p* = 0.045) compared to the sham treatment group.

## 4. Discussion

In the current study, we applied a transgenic MitoPark PD mouse model to study the effects of long-term probiotic treatment on motor symptoms. We found that the daily oral administration of probiotics for 16 weeks has neuroprotective effects and alleviates the progressive deterioration of motor functions in the MitoPark PD mice. Furthermore, the immunohistochemical staining results showed more preserved dopamine neurons in the probiotic-treated group than in the sham probiotic-treated group, suggesting a neuroprotective effect of probiotics. Up to now, the beneficial effects and the underlying mechanisms of probiotic treatment for PD have remained unclear. A PD animal model would be helpful in clarifying the benefits and underlying mechanisms of applying probiotics to PD. To the best of our knowledge, this is the first research study to confirm the long-term beneficial effects of probiotics on motor functions in the transgenic PD mouse model. It could be helpful in better understanding the mechanical insights of using probiotics for potential therapeutic possibilities in PD. 

The present study identified the effects of probiotics in the MitoPark PD model, which features neuronal dopamine loss caused by mitochondrial dysfunction as its characteristic pathology [31]. It has been reported the MitoPark mice can exhibit the cardinal features of PD, such as the adult onset of neurodegeneration, progressive deterioration of motor functions, GI dysfunctions, and gut microbiome changes [31,32,46]. Based on earlier studies, the decreased locomotor activity can be obviously seen at the age of 14 weeks. These signs then progress and start to show more phenotypic manifestations (e.g., abnormal locomotor function, tremor, and twitching) at 14–20 weeks of age in the MitoPark mice [31,40,47]. The moderately deteriorating clinical course makes MitoPark the right model for the current study. We, therefore, chose MitoPark mice as the model to assess the long-term effects of probiotics. 

In the current study, to validate the beneficial effects during probiotic treatment, we performed quantitative assessments on gait function, beam balance, and coordination, which are the common symptoms in PD patients. The results showed that daily administration of probiotic intervention for 16 weeks can significantly alleviate the balance, coordination, and gait impairments. Furthermore, compared with the sham probiotic treatment, we found that long-term probiotic treatment postponed the disease progression in PD mice. These data could enhance the growing amount of basic medical research and clinical application on the efficacy of probiotics in PD treatment. These results are paralleled with PD human studies showing the positive effects in MDS-UPDRS scores of PD patients after a 12-week administration of probiotics [29], encouraging further research into its therapeutic application. Other human studies have also shown several beneficial effects of probiotics in PD, including the alleviation of the symptoms in abdominal pain, bloating, and constipation and the improvements in stool consistency and defecation habits [28,48,49]. It has been increasingly reported that the gut microbial dysbiosis and alteration of microbial metabolites could play a role in the pathogenesis of PD. For example, the altered abundance of Bifidobacteriaceae, Christensenellaceae, Enterobacteriaceae, Enterococcaceae, Lachnospiraceae, Lactobacillaceae, Pasteurellaceae, Prevotellaceae, and Verrucomicrobiaceae families has been found in PD [50,51,52]. This dysbiosis of gut microbiota could explain several features of PD. Furthermore, the abundance *of* Lachnospiraceae and Enterobacteriaceae are related to disease severity, disease progression, and motor impairment in PD [53,54]. The lower levels of Lachnospiraceae and higher levels of Enterobacteriaceae families have been correlated with the severity of the disease, postural instability, gait difficulty, and motor impairment (assessed by MDS-UPDRS III and Hoehn and Yahr stage) [51,53]. In the current study, the mechanisms by which probiotics preserve several motor functions in PD are still not clear. The possible pieces of evidence supporting the efficacy of probiotics in PD could be related to the alternation of PD-associated microbiota composition, improvement of GI function, reduction of harmful gut bacteria, gut leakiness, bacterial translocation, proinflammatory cytokines, oxidative stress, potentially pathogenic bacterial overgrowth, and the associated neural inflammation in the ENS [24,25,26,27,30,55]. In addition, to enhance the effects of anti-inflammation and anti-oxidative stress in the GI system, combining the probiotics and other drug treatments (e.g., anandamide or calcitonin gene-related peptide) may have better effects to inhibit the inflammatory process and preserve the integrity of gastric mucosa, by improving the endogenous release of nitric oxide (NO) and increasing the blood flow [13,21,56,57,58,59].

Animal studies show that microbiota-based interventions can influence host physiology by modulating the function of mammalian neurotransmitters, such as dopamine, norepinephrine, and serotonin [60]. With respect to serotonin, it has been reported that more than 90% of serotonin is synthesized in the gastrointestinal tract by the enterochromaffin cells, which can be modulated by gut microorganisms via the production of short-chain fatty acids [61,62,63]. Similar to the dopaminergic denervation in PD, the loss of serotoninergic terminals was also found in PD [64]. Interestingly, manipulation of the microbiota can affect intestinal mobility by modulating dopamine or serotonin in the gut, as well as the turnover rate of dopamine and serotonin in the brain [65]. In an earlier animal study, it was found that probiotics have an effect on regulating the intestinal flora composition and the metabolism of serotonin [66]. However, the beneficial effects of probiotics on the serotonin level in PD are still unknown. Additional studies will be necessary for a complete understanding of the interaction of probiotics and serotonin and neurotransmitters.

The histological testing of tyrosine hydroxylase staining was performed in both groups and showed that more dopaminergic neurons were preserved in the group receiving probiotic treatment than in the sham control group. These results suggest that long-term probiotic administration may not only postpone the deterioration of motor impairments but also have neuroprotective effects against the progressive degeneration of dopaminergic cells in the SNpc. The present study, based on the animal behavioral and histological results, indicates that the early (starting at 8 weeks of age) and long-term (16 weeks) probiotic intervention may induce the preservation of several comprehensive motor functions through the protection of the nigrostriatal dopamine neurons. Similar neuroprotective effects have also been reported using oral administration of probiotics or fecal microbiota transplantation from normal mice in PD animal models [27,67,68]. The role of probiotics in ameliorating dopaminergic neuronal loss could be attributed to their actions of inhibiting glial cell activation and neuroinflammation, increasing butyrate level, and elevating the level of some neurotrophic factors such as brain-derived neurotrophic factor (BDNF) or glial cell line-derived neurotrophic factor (GDNF) [27,67,68]. Earlier studies have reported that the administration of probiotics increased the levels of BDNF and GDNF, which are required for the survival of dopaminergic neurons in vivo [67,69]. This could be one of the possible mechanisms that support our finding showing the neuroprotective effects in dopaminergic neurons following long-term treatment of probiotics. Another possible mechanism is the anti-inflammation effects of probiotics. It has been found that PD patients have greater intestinal permeability (gut leakiness), which might upregulate the local/systemic inflammatory response and oxidative stress induced by lipopolysaccharides (LPSs, also termed endotoxin), leading to α-synucleinopathy in the enteric nervous system [13]. Furthermore, the higher levels of gut-derived LPSs could promote the neuroinflammation and damage in substantial nigra via the disruption of blood–brain barrier (BBB) integrity [70]. The evidence on the effects of probiotics in anti-inflammation or LPS and their relation to PD is not known. It has been found that some specific probiotic bacteria (e.g., *B. longum* subsp. *Infantis*, *Lactobacillus fermentum* LAB9, or *L. casei* LABPC) may decrease colonic LPS concentrations and reduce the proinflammatory tone and decrease the LPS-induced neuroinflammation in in vitro and in vivo studies [71,72]. Because the underlying mechanisms are still not clear, further studies are still needed to clarify the mechanisms of action of probiotics in the neuroprotective effects for PD.

## 5. Conclusions

The current study provides a clearer picture of the changes in motor functions with disease progression in transgenic PD mice with and without probiotic treatment and documents the efficacy of probiotics in PD-related motor dysfunctions. Moreover, daily and long-term administration of probiotics may have a neuroprotective effect. Further preclinical research is still needed to clarify the underlying mechanisms, leading to improved therapeutic protocols of probiotics in PD or other neurodegenerative diseases.
